# The Effect of Post-Activation Potentiation Enhancement Alone or in Combination with Caffeine on Anaerobic Performance in Boxers: A Double-Blind, Randomized Crossover Study

**DOI:** 10.3390/nu16020235

**Published:** 2024-01-11

**Authors:** Yinkai Zhang, Penglin Diao, Jie Wang, Shiying Li, Qingmin Fan, Yunzhi Han, Yapu Liang, Ziyu Wang, Juan Del Coso

**Affiliations:** 1 China Wushu School, Beijing Sport University, Beijing 100084, China; 2 China Swimming College, Beijing Sport University, Beijing 100084, China; 3 Sports Coaching College, Beijing Sport University, Beijing 100084, China; 4 Wushu Instructor Training Base for International Promotion of Chinese Language, Beijing Sport University, Beijing 100084, China; 5 School of Humanities, Beijing Sport University, Beijing 100084, China; 6 School of Strength and Conditioning Training, Beijing Sport University, Beijing 100084, China; 7 Sport Sciences Research Centre, Rey Juan Carlos University, 28943 Fuenlabrada, Spain; juan.delcoso@urjc.es

**Keywords:** performance enhancement drug, ergogenic aid, combat sport, stimulation, athlete

## Abstract

Post-activation performance enhancement (PAPE) is a physiological phenomenon that refers to an acute excitation of the neuromuscular system following intense exercise that ends in enhanced physical performance in a subsequent bout of exercise. The scientific literature has primarily examined the effectiveness of PAPE alone or combined with caffeine (CAF) intake in all-out tests lasting ≤10 s, as the effect of PAPE is transitory. The aim of the present study was to determine the effect of a protocol to induce PAPE alone or in combination with caffeine intake on the 30 s Wingate Anaerobic Test in highly trained boxers. Twenty-five male and highly trained boxers (mean age: 20 ± 1 years) participated in a double-blind, randomized crossover study consisting of three different experimental conditions: (i) control (CON), with no substance intake and no PAPE protocol before the Wingate Anaerobic Test; (ii) PAPE + PLA, involving the intake of a placebo 60 min before and a PAPE protocol comprising a 10 s cycling sprint overloaded with 8.5% of the participants’ body weight 10 min before the Wingate Anaerobic Test; and (iii) PAPE + CAF, involving the intake of 3 mg/kg of caffeine 60 min before and the same PAPE protocol used in the (ii) protocol before the Wingate Anaerobic Test. In all conditions, the participants performed the 30 s version of the Wingate Anaerobic Test with a load equivalent to 7.5% of their body weight, while the cycle ergometer setting was replicated. Immediately following the Wingate test, heart rate (HR), the rating of perceived exertion (RPE), and blood lactate concentration (Bla) were measured. In comparison to CON, PAPE + PLA enhanced mean power (*p* = 0.024; Effect size [ES] = 0.37) and total work (*p* = 0.022; ES = 0.38) during the Wingate test, accompanied by an increase in post-test blood lactate concentration (*p* < 0.01; ES = 0.83). In comparison to CON, PAPE + CAF enhanced mean power (*p* = 0.001; ES = 0.57), peak power (*p* = 0.013; ES = 0.57), total work (*p* = 0.001; ES = 0.53), post-test blood lactate concentration (*p* < 0.001; ES = 1.43) and participants’ subjective perception of power (*p* = 0.041). There were no differences in any variable between PAPE + PLA and PAPE + CAF. In summary, a PAPE protocol that involves a 10 s all-out sprint 10 min before the Wingate Anaerobic Test was effective in enhancing Wingate mean power in highly trained boxers. The addition of 3 mg/kg of caffeine to the PAPE protocol produced an effect on mean power of a higher magnitude than PAPE alone, and it enhanced peak power along with participants’ subjective perception of power. From a practical point of view, PAPE before exercise seems to be an effective approach for increasing Wingate performance in highly trained boxers, while the addition of caffeine can increase some benefits, especially peak power.

## 1. Introduction

In recent years, sports practitioners have been looking for new methods to acutely improve athletes’ performance, for example, via the use of physical [1,2,3,4,5] and nutritional [6,7,8,9,10] strategies carried out moments before the onset of exercise. One of the more researched topics within the physical strategies performed before exercise to increase physical performance is the use of short and intense exercise protocols to produce a post-activation potentiation enhancement (PAPE) [11,12,13,14]. PAPE, in essence, is a physiological phenomenon that produces an acute excitation of the neuromuscular system following an intense exercise bout. With the appropriate time of recovery, the neuromuscular excitation may produce enhanced physical performance in a subsequent bout of exercise [15]. A large number of studies have confirmed the existence of PAPE, but they suggest that the exercise protocol to induce PAPE has to be of maximal (or near) intensity, while PAPE is primarily effective in enhancing short and high-intensity activities [16,17,18].

Evidence also suggests that one of the key factors in optimizing PAPE is that the exercise performed to induce neuromuscular excitation should mimic the movement patterns of the subsequent exercise bout [19,20]. For example, a recent investigation that used a 10 s all-out sprint against a load equivalent to 8.5% of participants’ body weight for the induction of PAPE showed a significant increase of 0.6% in peak power and 2.2% in mean power during the 30 s Wingate Anaerobic Test performed 10 min after the PAPE protocol [20]. However, the time of recovery between the PAPE protocol and the exercise bout is also crucial. For example, a cycling-based PAPE protocol lasting 20 min with exercise of progressive intensity and 2 × 20 s sprints tended to be more effective in enhancing cycling performance during a 4 min maximal-performance test when there was 20 min instead of 6 min of recovery [21]. Interestingly, the time of recovery between the PAPE protocol and the exercise bout may be dependent on the PAPE protocol, as it may be as short as 3 min if the PAPE protocol includes jumps and sprints with and without sled towing [22]. From a practical point of view, the efficacy of PAPE may rely on a net balance between fatigue and potentiation [23], suggesting that the exercise performed to induce PAPE has to be intense enough to produce neuromuscular excitation but there has to be enough time for recovery before the bout of exercise to avoid fatigue dampening the potential excitatory enhancement.

While PAPE on its own provides an attractive solution for coaches to acutely improve exercise performance, other methods may be synergistically applied to PAPE protocols, such as nutritional interventions. Among all the possible nutritional strategies to be combined with PAPE, caffeine seems the most promising as it may potentiate the excitatory effects of PAPE [22]. The effect of the acute intake of caffeine is one of the most frequently researched topics in the field of sports nutrition, as there is extensive research suggesting that caffeine enhances all-out exercise performance [24,25,26]. For example, a meta-analysis including 16 studies on the effect of caffeine on performance during the Wingate Anaerobic Test showed that caffeine significantly increased peak and average power [27]. Although there is plenty of evidence to support that both PAPE and caffeine intake enhance all-out exercise performance when used alone, studies about the synergy of combining PAPE protocols and caffeine intake are scarce and contradictory [22,28,29]. The addition of 5 mg/kg of caffeine to a PAPE protocol augmented the PAPE effect on jump performance in football players [22]. Likewise, the addition of 3 mg/kg of caffeine to a PAPE protocol augmented the PAPE effects on several taekwondo-specific exercise tests that include agility movements and kicking [28]. However, the addition of 6 mg/kg of caffeine to a PAPE protocol had no additional effect over PAPE on the jumping performance of volleyball players [29]. Beyond the scarcity of data, the aforementioned studies tested the combination of PAPE and caffeine in exercise tests of ≤10 s (i.e., jumping, agility tests, and 10 s kicking tests). However, there seems to be a gap in current research focusing on tests lasting more than 10 s. Given the variations in energy supply systems across different performance durations [30], it becomes evident that the assessment of exercise performance in a task lasting longer than 10 s is essential to expand understanding of the effects of PAPE alone or in combination with caffeine during short-term all-out exercise. Moreover, the 30 s Wingate test stands out as the “gold standard” to assess anaerobic capability as it correlates with performance in various sports events [31,32,33,34,35], encompassing disciplines such as running, cycling, skating, swimming, jumping, and combat sports. The widely recognized validity of the 30 s Wingate test makes it the best method for evaluating anaerobic capacity as its measurement involves the assessment of the adenosine triphosphate and phosphocreatine system (ATP-PCr; as peak power) and the glycolytic system (as mean power) [36].

Of all the athletes that may benefit from the effect of PAPE alone or in combination with caffeine, boxers are among the ones with a higher potential benefit as boxing is a sport characterized by short and explosive movements. Overall, a boxing match is characterized by intermittent bursts of high-intensity activity, interspersed with periods of low-intensity or paused activity due to holds by boxers or referee interruptions [37,38]. The action-to-rest ratio is approximately 3:1 [39]. This pattern of activity demands a substantial level of anaerobic capacity [40] to effectively meet the energy demands of the box matching. Moreover, it has been observed that while boxing is primarily aerobic in nature, crucial movements such as offensive scoring or knockout (KO) maneuvers rely predominantly on anaerobic metabolism [41]. In addition, several articles have been published showing that interventions in the lower body can improve upper body performance [14,42,43]. This suggests that improvements in lower body power, as measured by the Wingate test, might have the potential to translate into enhanced punching power for boxers. This might be the reason why many studies have measured Wingate performance among boxers [44,45,46].

For all the expressed above, the aim of the present study was to determine the effect of a protocol to induce PAPE alone or in combination with caffeine intake on the 30 s Wingate Anaerobic Test in highly trained boxers. We used boxers to fulfill this aim, as combat sports entail all-out movements and maneuvers with contributions from both aerobic [47] and anaerobic metabolism [48]. We hypothesized that the addition of caffeine to a PAPE protocol would induce an augmentation of the PAPE effect on Wingate performance.

## 2. Methods

### 2.1. Participants

A total of 30 male boxers from Beijing Sport University were recruited for this study. All participants recruited for the study were provincial- to national-level athletes, possessing a minimum of 4 years (5 ± 1 years) of boxing training experience. These individuals have achieved notable success, having won at least one provincial competition or secured a position within the top-5 ranking in a national competition. To control for individual differences in habitual responsiveness to caffeine due to tolerance, only participants with a daily caffeine intake of less than 50 mg/d were included [49]. We used only male boxers because the potential impacts of PAPE [50] may be influenced by gender-specific factors. This choice aimed to mitigate potential confounding variables associated with gender, ensuring a more focused examination of the targeted effects. In addition to daily caffeine intake, inclusion criteria were as follows: (i) absence of neuromuscular and musculoskeletal disorders, (ii) resistance training experience of at least 2 years, and (iii) self-described satisfactory health status. If participants reported (i) a positive smoking status or (ii) a potential allergy to caffeine, they were excluded from the experiment. Twenty-five male athletes met these inclusion/exclusion criteria (age: 20 ± 1 years; height: 178 ± 4 cm; body weight: 80 ± 12 kg) and completed all experiments. An efficacy analysis conducted using G*POWER 3.1.9.6 (University of Kiel, Germany) indicated that 24 participants were required for this study. The analysis assumed an efficacy of 0.80, an alpha level of 0.05, an effect size of 0.27, and a correlation of 0.5 between repeated measures. The chosen effect size was derived from previous meta-analyses comparing caffeine versus placebo on the Wingate test [27]. Each participant was familiar with sprint-based cycling exercises, and participants were allowed to withdraw from the experiment at any time. All participants were informed about the objectives and potential risks of the study before providing written informed consent for participation. The study protocol was approved by the Sports Science Experiments of Beijing Sport University (No. 2023161H) and was conducted in accordance with the ethical standards of the Declaration of Helsinki.

### 2.2. Study Design

**Figure 1:**
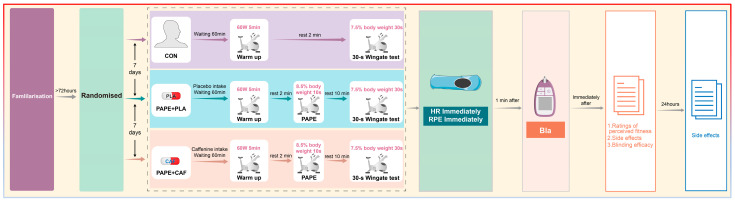
Experimental design. CON = control; PAPE = post-activation performance enhancement protocol; PLA = placebo; CAF = caffeine; HR = heart rate; RPE = rating of perceived exertion; Bla = blood lactate concentration.

Participants attended one familiarization session and three randomized experimental sessions over a ~3-week period, with each experimental session separated by one week (refer to Figure 1). The familiarization session was implemented at the beginning of the experiment and was primarily designed to obtain body characteristic assessments and to acquaint participants with the experimental procedure and the tests to be conducted, aiming to prevent any potential learning effect. Comfortable seat height and handlebar position on the cycle ergometer were set and recorded for later experimental sessions. At least 48 h after the familiarization session, the experimental sessions were conducted in a randomized order. The order of the trials for each participant was assigned using the randomization feature provided by the online software RANDOM.ORG [51].

The three conditions under experimentation were as follows: (i) control (CON), which involved no substance intake and no PAPE protocol before the Wingate Anaerobic Test; (ii) PAPE + PLA, which involved a placebo intake 60 min before and a PAPE protocol comprising a 10 s cycling sprint overloaded with 8.5% of the participants’ body weight 10 min before the Wingate test; and (iii) PAPE + CAF, the intake of 3 mg/kg of caffeine 60 min before and the same PAPE protocol used in the (ii) protocol 10 min before the Wingate test. (Figure 1). In all conditions, the participants performed a 5 min warm-up and then performed the 30 s version of the Wingate Anaerobic Test with a load equivalent to 7.5% of their body weight while the cycle ergometer setting was replicated. Immediately following the Wingate test, heart rate (HR) and the rating of perceived exertion (RPE) were measured, whereas blood samples were obtained 1 min after the Wingate test to assess blood lactate concentration (Bla). All trials were performed in a laboratory with controlled ambient temperature and humidity (24 ± 1 °C and 43  ±  2%, respectively). The primary outcomes for this study were power parameters derived from the Wingate test. Secondary outcomes involved pertinent physiological parameters such as HR, RPE, and Bla and other parameters such as subjective perceived fitness and prevalence of side effects.

### 2.3. Experimental Protocol

To minimize the impact of circadian rhythm disturbances, each testing session occurred within a consistent ~2 h time window relative to the first experimental test. Additionally, participants were instructed to refrain from engaging in any intense exercise 48 h prior to each test to avoid fatigue. Throughout the study, participants were advised to maintain their regular nutritional, sleep, and training habits and abstain from additional supplements, caffeine sources, and alcohol for 24 h preceding each testing session. A list of caffeinated foods and beverages (e.g., coffee, tea, soft drinks, energy drinks, cola beverages, chocolate drinks, and chocolate) was provided to all participants prior to the start of the experiment so that they could avoid caffeine from 24 h prior to the study until the end of the study [52].

In all trials, participants arrived at the laboratory 75 min before the start of the session and 2 h after the last meal. For CON, no capsule was given, and participants rested supine on a stretcher for 60 min. For PAPE + PLA and PAPE + CAF, participants were given a capsule containing either 3 mg/kg caffeine or a placebo and rested for 60 min. We set a time of 60 min between ingestion and the onset of exercise to produce peak blood caffeine concentrations during the testing [53]. The capsules containing caffeine or placebo were identical, and participants were unable to visually discern the ingredients within the capsules. The capsule contained either 300 mg maltodextrin (My Protein, Manchester, UK) as a placebo for the PAPE + PLA trial or 3 mg/kg caffeine (Nutricost, Vineyard, UT, USA) for the PAPE + CAF trial, and it was ingested with 100 mL of water. The dose of caffeine was chosen based on the ISSN position on caffeine [6] and based upon several investigations that have found an improvement in performance with such a dose.

After a 60 min rest period, participants underwent a standardized warm-up that consisted of 5 min at 60 W, followed by an unloaded 10 s sprint in the control condition (CON). After a 2 min period, participants were asked to perform the 30 s Wingate test. For the conditions of PAPE + PLA and PAPE + CAF, participants followed the same warm-up routine as in CON 60 min after substance intake but performed a 10 s maximal cycling sprint against a workload equivalent to 8.5% of their body weight, aiming to induce an excitatory enhancement. A period of 10 min of recovery after the PAPE protocol and the onset of the Wingate test was set to allow a net balance between fatigue and potentiation [23] as this 10 min recovery time was previously found to be effective in increasing Wingate performance after this same PAPE protocol [20].

A 10 min recovery period post-PAPE protocol and before the onset of the Wingate test was implemented to establish a net balance between fatigue and potentiation [21], considering the effectiveness of this 10 min recovery time in enhancing Wingate performance after a PAPE protocol [20]. The Wingate test was conducted using a Monark cycle ergometer (Ergomedic 894E, Vansbro, Sweden), involving 30 s of riding at maximum pedaling velocity with a load equal to 7.5% of the participant’s body weight [54]. Participants were instructed to pedal as fast as possible from the beginning to the end of the test in order to reach the maximum rpm in the shortest possible time and to try to maintain this pedaling speed until the end of the test. Standardized encouragement was given to the participants in all trials. One researcher verified that participants remained seated during the whole test, particularly during the initial phase of the sprint. Peak and minimum cycling power were used to calculate the Wingate fatigue index (%) as previously suggested [55,56,57]. Researchers were blinded to conditions during the gathering of data and calculation of performance data.

Just after the end of the Wingate test, participants were asked about their RPE using the 6–20-point Borg scale [58]. Peak HR was obtained just after exercise using a heart rate sensor chest strap (H9, Polar Electro Oy, Kempele, Finland) [59]. One minute after the end of the Wingate test, capillary blood samples were obtained from a fingertip to assess Bla with an enzymatic–amperometric device (Biosen C-Line; EKF Diagnostics, Barleben, Germany). The Biosen C-Line has excellent accuracy with a coefficient of variation of less than 2.0% [60], and it is widely used in the field of sports science for measuring athletes’ blood lactate concentrations [61,62,63]. Following the collection of blood samples, participants were requested to complete a questionnaire evaluating their subjective perceptions of power, endurance, and fatigue during the Wingate test. The questionnaire employed a 1- to 10-point scale for each item, with participants being informed beforehand that a score of 1 represented the lowest amount of the item, while a score of 10 indicated the highest amount [64]. Then, immediately after testing and 24 h post-testing, participants were instructed to complete a questionnaire assessing the effect of the conditions on sleep quality, feelings of nervousness, gastrointestinal issues, and any other discomforts using a yes/no scale [64,65]. Lastly, to evaluate the success of blinding, participants were required to guess in what trial they had received placebo and caffeine via the following question: “Which supplement do you think you have received today?”. They were provided with three response options: “caffeine”, “placebo”, or “I don’t know”.

### 2.4. Statistical Analysis

Data are expressed as mean ± standard deviation (M ± SD) for all variables. Shapiro–Wilk tests were used to determine whether all variables were normally distributed, which was confirmed with *p* values above 0.05 in all cases except participants’ subjective perception of power, RPE, and exertion. To assess differences in Wingate performance variables, post-exercise HR and Bla, and self-perceived performance after exercise among trials (i.e., CON, PAPE + PLA, and PAPE + CAF), a one-way repeated measures analysis of variance (ANOVA) with Bonferroni correction was conducted. Partial eta squared (η^2^p) was used as the effect size for the one-way repeated ANOVA [66]. For participants’ subjective perception of power, RPE, and exertion, Friedman’s tests were utilized. Post hoc tests were then performed employing the Wilcoxon signed-rank test. Additionally, to compare the prevalence of side effects after exercise and 24 h after, we employed the Cochran Q test due to the dichotomous nature of the data. The practical significance of the effects of PAPE + PLA and PAPE + CAF in comparison to CON was assessed by calculating Cohen’s *d* effect sizes (ES) [67]. This analysis was specifically applied to outcomes that met the assumptions of normality and continuity for continuous variables. Cohen’s *d* greater than 0.8 was considered large, between 0.8 and 0.5 was categorized as medium, between 0.5 and 0.2 was considered small, and less than 0.2 was deemed insignificant [68]. The success of the blinding procedures was examined using Bang’s blinding index, as previously suggested [69]. Statistical analyses were performed using SPSS (version 26.0; SPSS, Inc., Chicago, IL, USA), and the significance level was set at *p* ≤ 0.05.

## 3. Results

### 3.1. Wingate Test and Subsequent HR, RPE, and Bla Measurements

The results of the three conditions regarding Wingate performance and post-exercise HR, RPE, and Bla are summarized in Table 1. There was a main effect of the condition on peak power, mean power, total work, and Bla (*p* < 0.05). Post hoc comparisons with Bonferroni correction unveiled higher values for mean power, total work, and Bla in both PAPE + PLA and PAPE + CAF conditions compared with CON. These performance variables exhibited larger increases in the comparison PAPE + CAF vs. CON (ES = moderate) than in the comparison PAPE + PLA vs. CON (ES = small). Moreover, in the case of peak power, only PAPE + CAF exhibited a significant enhancement over CON (*p* = 0.013). No significant differences were observed in any of the performance variables between the PAPE + PLA and PAPE + CAF conditions (*p* > 0.05).

### 3.2. Perceived Fitness, Side Effects, and Blinding Efficacy

Table 2 presents the subjective feelings of power, endurance, and fatigue, as well as the occurrence of side effects after exercise and 24 h after across the three conditions. No statistically significant differences were found between the three conditions in any particular aspect (*p* > 0.05), except in self-perceived power, where participants reported higher values after PAPE + CAF than after CON (*p* = 0.041). In the PAPE + CAF condition, 11 participants correctly guessed that they had ingested caffeine (Bang Index = −0.08), while in the PAPE + PLA condition, 13 participants correctly guessed that they had ingested the placebo (Bang Index = 0.16). These results suggest the successful blinding of participants to the substance intake.

## 4. Discussion

It was demonstrated that caffeine intake in a dose of 3–5 mg/kg, in conjunction with a PAPE protocol including plyometrics and heavy-weight squatting, led to a synergistic ergogenic effect in jumping performance, agility, and kicking [22,28]. However, there is no study that has been geared toward determining the effect of adding caffeine to a PAPE protocol that mimics the subsequent activity (i.e., cycling) on an exercise performance test lasting > 10 s. The findings from the present study reveal that both PAPE + PLA and PAPE + CAF conditions resulted in significantly higher values of mean power and total work during the 30 s Wingate test with respect to CON. Although there were no statistically significant differences between PAPE + PLA and PAPE + CAF for any performance variable, the magnitude of the ergogenic effect was always higher with PAPE + CAF than with PAPE + PLA. Additionally, only the PAPE + CAF condition increased peak power in the Wingate test and the subjective perception of power. Collectively, these data suggest that a PAPE protocol that involves a 10 s all-out sprint 10 min before the Wingate Anaerobic Test was effective in enhancing Wingate mean power in highly trained boxers. Although there was no synergistic effect of combining PAPE and caffeine intake, the addition of 3 mg/kg of caffeine to the PAPE protocol was the only protocol that increased peak power during the Wingate test and produced benefits of higher magnitude than PAPE alone. From a practical standpoint, boxing practitioners may consider the use of a PAPE protocol as an effective strategy to enhance Wingate performance, while the addition of caffeine may augment the benefits of PAPE. Lastly, either PAPE alone or combined with caffeine intake produced any measurable side effects after exercise or 24 h after, which suggests their utility to be used safely in the context of sports performance.

Consistent with prior research, the conditioning activity performed in this study to induce PAPE (i.e., a heavy-loaded 10 s all-out sprint) was effective in enhancing subsequent exercise performance when setting an appropriate time of recovery (i.e., 10 min) [20]. Although the current study did not test different recovery times between PAPE and the bout of exercise, a previous study demonstrated that the addition of caffeine to a PAPE protocol may reduce the recovery time to obtain ergogenic benefits with PAPE [22]. Not only was there enough recovery time but the current investigation was also designed to ensure that the PAPE protocol mimicked the exercise to be performed afterward (cycling sprints in both cases). In this context, we found that the PAPE protocol was effective in enhancing Wingate performance with and without caffeine. These data imply that using a conditioning activity that includes a cycling sprint is effective in inducing PAPE on a subsequent all-out test, as previously found [21]. This is consistent with previous research that found that utilizing a conditioning activity to induce PAPE mirroring subsequent test movements is effective in obtaining ergogenic benefits [19,70,71,72]. The novelty of the current investigation is the suggestion that the addition of caffeine intake (60 min before) to a PAPE protocol with appropriate recovery time and mimicking the exercise to be performed afterward may induce some “extra” performance benefits, particularly to enhance peak power output and the athletes’ subjective perception of power. Taken together, these studies suggest that the conditioning activity to induce PAPE has to be carefully designed to allow for recovery and to replicate the movements of the “target” exercise, while caffeine can be considered an accessory strategy if the performance situations demand peak power output.

The net balance of fatigue and potentiation factors when employing PAPE has been explored in some studies [23,73], with previous research indicating a delay in the onset of potentiation, especially after employing heavily intense exercise [74,75]. This delay may be attributed to an initial fatigue effect that may be less pronounced with the use of specific activities (plyometric exercise) with respect to a general protocol of resistance exercise when the objective is to increase jump and sprint performance [76,77]. While studies examining conditioning activities that mirror subsequent test movements in jumping, swimming, and running have not determined 10 min as the optimal recovery time [19,70,71,72], the current investigation suggests that this time may be optimal for all-out cycling sprints of relatively short duration (10 s for the PAPE and 30 s for the target exercise bout). Interestingly, a study by Christensen and Bangsbo [21] revealed a significant decrease in cycling mean power with a recovery time of only 6 min after the PAPE protocol with respect to the same PAPE protocol performed with 20 min of recovery. Although much investigation is still needed to understand optimal recovery times for PAPE, current data emphasize the importance of determining the optimal recovery period based on the program and intensity employed to induce PAPE.

The current study also noted increased blood lactate concentrations after the Wingate test for both PAPE + PLA and PAPE + CAF. These findings align with prior research suggesting that PAPE’s ergogenic effect is accompanied by an increased accumulation of blood lactate [78]. This is somewhat expected, as increased performance during the Wingate test is habitually accompanied by greater post-exercise concentrations of lactate [24,79]. This may be related to the phosphorylation of myosin-regulated light chains and the activation of more type II muscle fibers, thereby increasing lactate concentration via anaerobic metabolism [80]. Briefly, the main energy pathway to produce energy for muscle contraction during the Wingate test is glycolysis and the higher post-exercise blood concentration of lactate with PAPE likely indicates a higher absolute glycolytic contribution during the Wingate with PAPE. Although it is a hypothesis pending confirmation, we suggest that the effect of PAPE on the 30 s Wingate test may be mediated or linked by an improved glycolytic contribution.

In the current study, we employed a moderate dose of caffeine intake (3 mg/kg) combined with PAPE, which reported several performance benefits such as better peak power, mean power, and total work compared to the control condition. Notably, when compared to PAPE alone, the combined use of caffeine supplementation and PAPE yielded larger effect sizes in the mentioned performance variables. These outcomes align with previous studies highlighting the capacity of caffeine intake to enhance anaerobic performance [24,25]. However, the lack of differences between PAPE alone and PAPE + CAF suggests that, in this study, the potential of caffeine to enhance Wingate performance was diminished with respect to the effect found when caffeine is ingested alone [24,25,79]. This may be due to the excitatory nature of both protocols, as PAPE and caffeine intake produce their ergogenic benefits via better neuromuscular excitation [81]. In this context, it seems that the use of either caffeine alone or PAPE alone may induce benefits for Wingate performance; the combination of both protocols may not produce a synergistic or additive effect due to the coincidence in the mechanism of action, but they may represent the best option when trying to reach the best performance [22,28,29].

In addition, caffeine intake may reduce the perception of fatigue during high-intensity muscle contractions, as it can block adenosine receptors, leading to a loss of adenosine’s “fatiguing” effect on the central nervous system [82]. This reduction in adenosine’s influence may contribute to a decrease in RPE and pain perception [83]. Given that the RPE scale was designed to correlate with heart rate response to aerobic exercise and the perceived exertion level [84], the absence of a significant difference in RPE may not be surprising in this study, especially considering that caffeine did not seem to alter heart rate after the Wingate test. Moreover, it is worth noting that the effect of caffeine intake on perceived fatigue appeared to diminish with escalating exercise intensity [85]. Additionally, the Wingate test involves maximal intensity efforts, and the caffeine intake may elevate the intensity during the Wingate test. Under such circumstances, the lack of a decrease in RPE is reasonable, as the heightened anaerobic power and intensity in the Wingate test, induced by caffeine, may counteract the expected reduction in perceived exertion. Collectively, all this information suggests that the likelihood of caffeine intake enhancing Wingate anaerobic power in this study via a reduction in the perception of fatigue appears to be improbable.

Boxers also reported enhanced self-perceived power during the PAPE + CAF condition (Table 2), although subjective perceptions of endurance and exertion remained unchanged among the three conditions. Prior studies have noted improvements in feelings of power following caffeine intake on similar topics [28,86,87]. In addition to heightened vigor, most investigations have reported a higher incidence of side effects (e.g., activation and insomnia) in the hours following the ingestion of caffeine [88,89]. However, in the current study, the incidence of related side effects was similar across three conditions among the boxers. This could be attributed to the caffeine dosage and the circadian timing of caffeine intake, which was set in the morning for most participants. The occurrence of side effects due to a caffeine dosage of 3 mg/kg and caffeine intake in the morning and midday was relatively low [64,90]. As a practical application, it can be suggested that the use of caffeine to enhance performance should be planned for exercise sessions performed in the morning, when possible, to reduce the side effects of this nutritional intervention.

The findings presented in this study bear relevance to boxers and athletes involved in sports characterized by high power output, given the high correlation between the Wingate test and other predominantly “anaerobic” sports events [31]. However, several limitations of the current study should be discussed to understand the scope of the study outcomes. These limitations include the study of a singular dose of caffeine and the employment of a cycling test for boxers, as no boxing-specific tests have been conducted. It is recommended to explore the different benefits obtained with different caffeine doses added to PAPE, such as 3 or 6 mg/kg of participants’ body mass, considering the potential dose–response effect of caffeine [91]. Additionally, the present study did not control for potential variations in the optimal rest period after the PAPE protocol when synergized with caffeine intake. Investigating the impact of multiple time-point combinations of caffeine intake in conjunction with a PAPE protocol in future investigations would contribute to the understanding of the best combination of these two protocols to maximize their effect on performance. Furthermore, this study did not include a “caffeine only” trial [caffeine (+) + PAPE (−)], which represents a potential limitation as it impedes determining if there was an additive/synergistic effect to PAPE and caffeine in the PAPE + CAF trial. Finally, the exclusive measurement of physiological parameters immediately after the Wingate test may be considered a limitation as we did not study the effect of the protocols on the time of recovery after the Wingate test. In essence, while this study provides valuable insights, the interpretation of immediate post-exercise physiological parameters and the absence of certain conditions should be considered within the constraints of the current experimental design. Future studies could enhance understanding of the effects of caffeine and PAPE on performance by conducting assessments of HR, RPE, and Bla at multiple time points. Measurements taken before, during, and after exercise would provide a more nuanced insight into the dynamic impact of caffeine and PAPE on the physiological parameters associated with Wingate performance in this sample of male boxers.

## 5. Conclusions

This study suggests that an easy-to-apply PAPE protocol identical to the subsequent testing maneuvers (i.e., cycling) emerges as an effective method in enhancing mean power and total work in the Wingate test in highly trained boxers. The addition of caffeine to the PAPE protocol produced further benefits as it increased the magnitude of the effect on mean power and was the only protocol that increased peak power along with the subjective perception of power. Moreover, the ingestion of caffeine with the PAPE protocol did not lead to adverse side effects, such as insomnia. Collectively, all these outcomes suggest that the combination of PAPE and caffeine may be a strategy to enhance short-term all-out performance in boxers, although further investigations are warranted to widen the scope of the potential benefits of this strategy in other sports, in women athletes and in high-intensity intermittent exercise performance.

## Figures and Tables

**Table 1:** Performance variables during a 30 s Wingate Anaerobic Test and post-exercise heart rate (HR), rating of perceived exertion (RPE), and blood lactate concentration (Bla). The Wingate test was performed after a control (CON) condition or after a post-activation performance enhancement protocol without caffeine (PAPE + PLA) or with caffeine (PAPE + CAF).

Variable (Units)	CON	PAPE + PLA	PAPE + CAF	*p*	ES		
					η^2^p	Cohen’s *d* CON vs. PAPE + PLA	Cohen’s *d* CON vs. PAPE + CAF
PP (W)	768.57 ± 128.27	809.74 ± 112.55	839.12 ± 140.64 *	**0.006**	0.189	0.34 (small)	0.52 (moderate)
MP (W)	564.31 ± 105.30	600.98 ± 90.45 *	622.33 ± 110.31 *	**<0.001**	0.280	0.37 (small)	0.54 (moderate)
TW (J)	16,314.64 ± 3166.64	17,411.64 ± 2654.70 *	18,043.68 ± 3360.08 *	**<0.001**	0.275	0.38 (small)	0.53 (moderate)
FI (%)	56.04 ± 11.52	55.73 ± 10.14	55.21 ± 11.93	0.945	0.02	−0.03 (very small)	−0.07 (very small)
RPE (arbitrary units)	17.20 ± 1.32	17.36 ± 0.91	17.64 ± 0.86	0.399	—	—	—
HR (bpm)	182 ± 4	184 ± 6	185 ± 7	0.126	0.083	0.41 (small)	0.42 (small)
Bla (mmol.L^−1^)	12.80 ± 1.77	14.34 ± 1.93 *	15.52 ± 2.03 *	**<0.001**	0.592	0.83 (large)	1.43 (large)

Data are presented as mean ± standard deviations. CON = control; PAPE = post-activation performance enhancement protocol; PLA = placebo; CAF = caffeine; ES = effect size; PP = peak power; MP = mean power; TW = total work; FI = fatigue index; * = significantly greater than CON condition (*p* < 0.05).

**Table 2:** Ratings of perceived fitness measured after a 30 s Wingate Anaerobic Test and prevalence of side effects immediately after and 24 h after the test. The Wingate test was performed after a control (CON) condition or after a post-activation performance enhancement protocol without caffeine (PAPE + PLA) or with caffeine (PAPE + CAF).

Items (Units)	CON		PAPE + PLA		PAPE + CAF		*p* for after	*p* for 24 h after
	Just after	24 h after	Just after	24 h after	Just after	24 h after		
Subjective feeling of power (arbitrary units)	5.60 ± 1.55	—	5.84 ± 1.49	—	6.40 ± 1.47 *	—	0.086	—
Subjective feeling of endurance (arbitrary units)	5.36 ± 1.63	—	5.32 ± 1.55	—	5.68 ± 1.77	—	0.669	—
Subjective feeling of fatigue (arbitrary units)	6.68 ± 1.46	—	6.88 ± 1.39	—	6.96 ± 0.93	—	0.441	—
Abdominal/gut discomfort (%)	8%	4%	4%	16%	12%	16%	0.472	0.276
Muscle soreness (%)	28%	76%	44%	76%	48%	80%	0.331	0.867
Increased urine output (%)	0%	8%	0%	4%	0%	12%	0.368	0.549
Headache (%)	0%	16%	0%	16%	0%	20%	0.999	0.895
Anxiety or nervousness (%)	0%	12%	0%	4%	4%	8%	0.368	0.607
Insomnia (%)	—	16%	—	12%	—	24%	—	0.529

The data are presented as mean ± standard deviations for continuous variables and as percentages (%) for binary categorical variables of side effects. CON = control; PAPE = post-activation performance enhancement protocol; PLA = placebo; CAF = caffeine. * = significantly greater than CON (*p* < 0.05).

## Data Availability

Data supporting the findings of this study can be requested from the corresponding author (Z.W.) upon reasonable justification. The data are not publicly available due to privacy concerns related to the inclusion of sensitive personal information.
